# A retrospective assessment of the KLIK PROM portal implementation using the Consolidated Framework for Implementation Research (CFIR)

**DOI:** 10.1007/s11136-020-02586-3

**Published:** 2020-08-15

**Authors:** Hedy A. van Oers, Lorynn Teela, Sasja A. Schepers, Martha A. Grootenhuis, Lotte Haverman, the ISOQOL PROMs and PREMs in Clinical Practice Implementation Science Group

**Affiliations:** 1 Psychosocial Department, G8-136, Emma Children’s Hospital/Amsterdam UMC, University of Amsterdam, Meibergdreef 9, Postbox 2266, 1100 DD Amsterdam, The Netherlands; 2 Princess Máxima Center for Pediatric Oncology, Utrecht, The Netherlands

**Keywords:** Implementation science, PROMs, Clinical practice, Framework

## Abstract

**Purpose:**

The KLIK Patient-Reported Outcome Measure (PROM) portal is an evidence-based intervention implemented in clinical practice in > 25 Dutch hospitals for patients (children and adults) who regularly visit the outpatient clinic. Implementation science frameworks can be used to understand why implementation succeeded or failed, to structure barriers and enablers, and to develop implementation strategies to overcome barriers. This paper aimed to (A) retrospectively describe determinants of successful KLIK PROM implementation using the Consolidated Framework for Implementation Research (CFIR), and (B) identify current barriers and match implementation strategies.

**Methods:**

(A) The KLIK implementation process was described retrospectively based on literature and experience, using the 39 CFIR constructs organized in five general domains: intervention characteristics, outer setting, inner setting, characteristics of individuals, and implementation process. (B) The CFIR-Expert Recommendations for Implementing Change (ERIC) Implementation Strategy Matching tool identified current barriers in the KLIK implementation and matched implementation strategies that addressed the identified barriers.

**Results:**

(A) The most prominent determinants of successful KLIK PROM implementation lie in the following CFIR domains: intervention characteristics (e.g., easy to use), characteristics of individuals (e.g., motivation), and process of implementation (e.g., support). (B) 13 CFIR constructs were identified as current barriers for implementing the KLIK PROM portal. The highest overall advised ERIC strategy for the specific KLIK barriers was to identify and prepare champions.

**Conclusion:**

Using an implementation science framework, e.g., CFIR, is recommended for groups starting to use PROMs in clinical care as it offers a structured approach and provides insight into possible enablers and barriers.

**Electronic supplementary material:**

The online version of this article (10.1007/s11136-020-02586-3) contains supplementary material, which is available to authorized users.

## Introduction

Patient-Reported Outcome Measures (PROMs) are standardized, validated questionnaires that are completed by patients, such as a person’s perspective on their health, well-being, or symptoms [1, 2]. PROMs can be used for several purposes: at group level to study differences between disease populations, to describe the effects of treatment in clinical trials, and to assess quality of care or on an individual level to promote patient-centered care, guide clinical decision-making, and to facilitate communication [3]. There is widespread evidence for the effects of PROM applications on an individual level regarding an increase in Health-related Quality of Life (HRQOL) scores, satisfaction with care and communication about PROs in research settings, both in adult [4–6] and pediatric [7–12] samples. Yet the implementation of these evidence-based (EB) PROMs interventions is challenging.

**Fig. 1:**
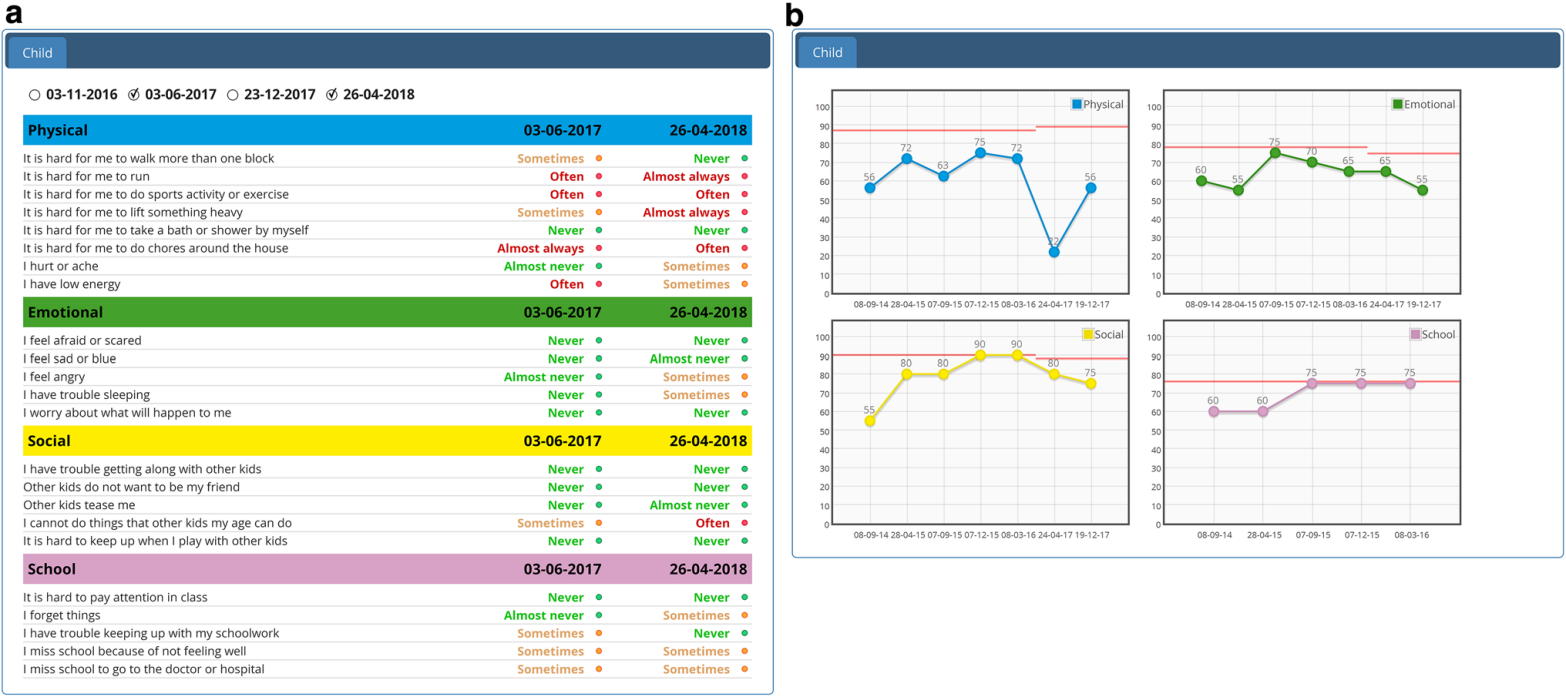
**a** KLIK ePROfile—literal feedback of the individual items on the Pediatric Quality of Life Inventory (PedsQL) **b** KLIK ePROfile—graphical feedback of the PedsQL, including norm lines

The KLIK PROM portal (www.hetklikt.nu and www.klik-uk.org) is an example of an EB PROM intervention for patients (children or adults) who regularly visit the outpatient clinic [13]. Patients complete PROMs online, prior to their visit. Answers are transformed into an electronic PROfile (ePROfile; Fig. 1). Clinicians discuss this ePROfile with patients, to monitor well-being over time, identify problems, and provide tailored advice and interventions. The effects of using the KLIK PROM portal have been demonstrated in pediatric oncology [7] and in pediatric rheumatology [12], by showing an increased and more detailed discussion of HRQOL and psychosocial functioning during the consultation, less undetected problems, and a higher clinician-reported satisfaction with provided care, without lengthening the consultation duration.

Despite the availability of several EB PROM interventions across the world, the actual implementation of PROM interventions in clinical practice remains limited [14–17]. There is a critical gap in behavioral medicine between what we know can optimize patient health and care outcomes and what gets implemented in everyday practice [1]. If EB PROM interventions are not successfully implemented in clinical practice, then intended effects are not reached, which limits the impact on patients’ health outcomes [18, 19]. Traditional randomized controlled trials study the effectiveness of PROM interventions under ideal circumstances. Yet for the implementation of PROMs in clinical practice, a different, more flexible approach is needed. Often, a “voltage drop” (a dramatic decrease in effectiveness) is seen once interventions get implemented in clinical practice [20]. Implementation research is defined by the National Institute of Health as the “scientific study of the use of strategies to adopt and integrate evidence-based health interventions into clinical and community settings in order to improve patient outcomes and benefit population health” [21]. Therefore, a scientific approach to the change process is crucial. In order to know what drives successful implementation of PROMs in clinical practice, we need to study the mechanisms that influence implementation outcomes [17, 22, 23]. Implementation science models, theories, or frameworks support in identifying factors that influence an implementation process or outcome.

In general, three overarching aims of theoretical approaches and five *categories* of theories, models, and frameworks used in implementation science can be distinguished [24]: (1) guiding the process of translating research into practice (*process models*), (2) understanding and/or explaining what influences implementation outcomes (*determinant frameworks, classic theories*, and *implementation theories*), and (3) evaluating implementation (*evaluation frameworks*). Specifically, determinant frameworks are useful in understanding or explaining what influences implementation outcomes and to support the design of implementation strategies or maximizing the use of enablers to implementation [24].

A widely cited and comprehensive determinant framework in the implementation science literature in health is the Consolidated Framework for Implementation Research (CFIR). Damschroder et al. [22] aimed to develop a framework that comprises common constructs from published implementation theories and includes, therefore, missing key constructs in other theories. It contains 39 constructs which are organized in five general domains: (1) intervention characteristics (e.g., evidence, complexity, adaptability, costs), (2) outer setting (e.g., peer pressure and external policies), (3) inner setting (e.g., structural characteristics, implementation climate, and culture), (4) characteristics of individuals (e.g., knowledge about the intervention and self-efficacy), and (5) implementation process (e.g., planning, engaging stakeholders, champions, and execution), see Fig. 3. Determinant frameworks, such as CFIR, are specifically useful in understanding or explaining what influences implementation outcomes and to support the design of implementation strategies or maximizing the use of enablers to implementation [24]. This paper aimed to (A) retrospectively describe the most prominent determinants and reasons of successful KLIK PROM implementation using CFIR and (B) use the CFIR-ERIC Implementation Strategy Matching tool to identify *current* barriers of the KLIK PROM portal implementation and match implementation strategies that address the identified barriers. In our specific study context, the CFIR framework seemed particularly useful as it covers a wide range of implementation constructs and domains and it allowed us to use a standardized framework to explain the influence of each domain on the implementation outcomes of an evidence-based PROM portal. With years of experience in the development and implementation, the KLIK PROM portal is now in a phase of understanding what barriers and facilitators have already been resolved and determining what major determinants are currently of influence to move to the next area of implementation: sustainability.

## Methods

### The evidence-based KLIK PROM portal

The development and implementation of the KLIK PROM portal is based on multiple studies (Supplemental Table 1). The predecessor of the KLIK ePROfile was the QLIC-ON PROfile [25]. During the QLIC-ON study, two generic HRQOL questionnaires widely used in pediatrics (TAPQOL [26] and PedsQL [27]) were converted into digital questionnaires. Patients were asked to complete a HRQOL questionnaire on a laptop in the waiting room of the outpatient clinic, prior to the visit. The literal answers and graphs were printed out, fed back to the pediatrician in a QLIC-ON PROfile on paper, and discussed with patients and parents during the consultation [25]. However, completing PROMs at the outpatient clinic and providing hard copy PROfiles was logistically complicated, and therefore, they are hard to implement in a real-world situation. As a result, the KLIK website (www.hetklikt.nu) was developed during the KLIK study in pediatric rheumatology [28]. From that moment, children and parents completed the questionnaires online at home. The implementation of KLIK, as part of standard care, started in 2011 [7, 12]. To gain more insight into the implementation process and outcomes, a study was conducted to identify barriers and enablers in this process in pediatric oncology [29].

**Table 1:** Description of the most prominent determinants of successful KLIK implementation using CFIR

CFIR domain	CFIR determinants	Reasons for successful implementation
Intervention characteristics	Evidence Strength & Quality	Effectiveness studies showed that KLIK is acceptable, valuable, and feasible [7, 12] The evidence of KLIK is emphasized in the training for clinicians [34]
Intervention characteristics	Trialability	KLIK started small and has found its way, step by step, in many hospitals and has scaled up to adult healthcare and other countries A license agreement is signed at the start, which can be ended and therefore undo the implementation if needed The implementation process and workflow are adapted according to the wishes of every multidisciplinary team, as the KLIK team experienced that a ‘one size fits all’ approach was not feasible
Intervention characteristics	Design Quality and Packaging	Clear and direct available feedback of PROMs on a well-designed dashboard The design of the KLIK PROM portal is evaluated positively, both by clinicians and patients [35] A strength of KLIK is the design of the PROM feedback and the variety of options [36] Optimization of the PROM feedback in KLIK is an ongoing process, based on scientific knowledge [37] and user experience
Outer setting	Cosmopolitanism	Worldwide, there is increased motivation for the use of PROMs in clinical practice, e.g., Value-Based Healthcare supports the use of PROMs, which facilitates the implementation climate The KLIK expert team shares common experiences with other hospitals through collaborations and networks (e.g., ISOQOL, PROMIS, research projects, implementation in many Dutch hospitals and the UK). Therefore, the KLIK PROM portal is increasingly well known and more visible for interested stakeholders
Outer setting	External Policy & incentives	Former research showed lack of formal agreements, such as policy and work plans on using KLIK at a hospital level [29]. However, this is changing, because from a governmental perspective, collecting PROMs or using Routine Outcome Monitoring for benchmarking purposes is increasingly encouraged or even obligated
Inner setting	Goals and feedback	During the KLIK training goals on implementing PROMs are clearly communicated, as previously different expectations were noticed (e.g., discussing PROMs in the consultation room versus collecting PROMs for research purposes), which may hinder the implementation Clinicians receive feedback regarding the implementation process during the annual evaluation meetings
Characteristics of individuals	Knowledge & Beliefs about the intervention	Multidisciplinary teams initiate implementation themselves and are, therefore, motivated to use KLIK. However, some clinicians of a team may have a negative attitude and show resistance, because they do not know the added value of using PROMs in clinical practice. The KLIK training provides knowledge of underlying principles and helps to generate enthusiasm Research shows that clinicians are more satisfied about their provided care when using PROMs [35] and that the majority of clinicians experience personal benefit from using KLIK, e.g., by helping them in communicating with patients/parents [36]
Characteristics of individuals	Self-efficacy	The KLIK training provides clinicians with knowledge, tools, and skills to feel competent to implement KLIK in their practice. However, there could even be more emphasis on training communication skills, as some clinicians report low confidence in discussing psychosocial topics with their patients Research shows that most clinicians have sufficient knowledge to use KLIK as intended [29] Current focus is on empowering patients to discuss PROMs with their clinician, for example by developing educational videos

**Fig. 2:**
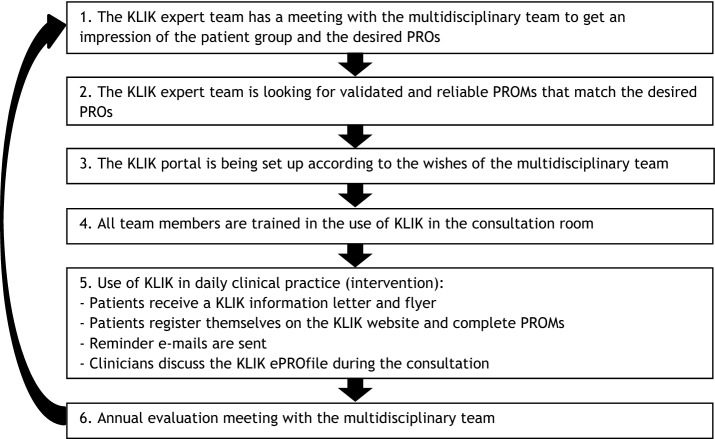
Overview of the KLIK implementation process for one multidisciplinary team. * The KLIK expert team consists of researchers with expertise in the field of (implementing) PROMs and HRQOL research

Currently, KLIK is part of standard care in > 70 different patient groups (e.g., diabetes, nephrology) in > 20 centers in the Netherlands and 3 centers in the United Kingdom. Over 17,000 patients are registered on the KLIK website and around 1,000 clinicians (e.g., physicians, nurses, psychologists) have been trained in the use of KLIK. KLIK is implemented in various settings, including hospital outpatient clinics, rehabilitation centers, and recently in dentistry. KLIK was initially developed for use in pediatrics, but since 2017 KLIK has also been implemented in adult care (e.g., coagulation diseases and medical psychology). The KLIK expert team of the Emma Children’s Hospital Amsterdam UMC coordinates the implementation of the KLIK PROM portal in pediatrics and adult healthcare in 20 hospitals in the Netherlands. The KLIK expert team in the Princess Máxima Center for pediatric oncology coordinates the implementation of KLIK in this center. KLIK can be implemented for any patient group, on request of a multidisciplinary team. The implementation procedure of the KLIK PROM portal has previously been described according to the guidelines of the International Society for Quality of Life Research (ISOQOL) [13]. A core element of the KLIK implementation process is to train all team members in the use of KLIK and discussing PROMs in the consultation room. A summary of the implementation process is shown in Fig. 2.

### Design

To retrospectively assess the KLIK PROM implementation using the CFIR framework, a mixed methods design was used. Part A consisted of a qualitative description regarding the most prominent determinants of successful KLIK PROM implementation. Part B consisted of an evaluation of current barriers in the KLIK implementation process and matching potential future strategies to reduce these barriers using the CFIR-ERIC Implementation Strategy Matching tool v1.0 [30, 31] and a qualitative description of the identified barriers and strategies that have been used already by the KLIK expert team.

### A. Retrospectively describing the most prominent determinants of successful KLIK PROM implementation using CFIR

The CFIR framework was used to retrospectively describe the implementation process of the KLIK PROM portal in different patient groups and hospitals throughout the Netherlands and to identify determinants in this process. Only the determinants relevant for the KLIK implementation process were described. To define which determinants were relevant for successful KLIK PROM implementation the following steps were taken. First, the KLIK PROM implementation process was described and discussed by the KLIK expert team, using all 39 CFIR constructs. However, for the reason of clarity, only the most prominent CFIR determinants relevant for the KLIK PROM implementation were extracted here (see Table 1). Second, the authors discussed which facilitators they found most prominent to describe. If the majority of authors considered a CFIR construct as valuable, it was included in the qualitative description. The KLIK implementation process was described based on published literature regarding the development, effectiveness, and implementation of KLIK in various settings and options for visual feedback of the PROMs (Supplemental Table 1) and unpublished literature (e.g., the KLIK user manual and training) about the KLIK portal and on experiences of the KLIK expert team.

### B. CFIR-ERIC Implementation Strategy Matching Tool to identify *current* barriers of the KLIK PROM portal implementation

The CFIR-ERIC Implementation Strategy Matching tool v1.0 [31] was used to identify current barriers in the ongoing KLIK implementation and to match implementation strategies that address the identified barriers. The CFIR-ERIC tool is based on the CFIR framework and the 73 Expert Recommendations for Implementing Change (ERIC) implementation strategies [32]. During the development of this tool [30], implementation researchers and clinicians (panelists) were presented with brief descriptions of barriers based on CFIR construct definitions. They were asked to rank implementation strategies that would best address each barrier.

Within the provided Excel tool, one can indicate which CFIR constructs are barriers to implementation. Five KLIK expert team members based in the Emma Children’s hospital Amsterdam UMC and three in the Princess Máxima Center for pediatric oncology involved in the implementation of the KLIK PROM portal independently indicated which of the 39 CFIR constructs were perceived as *current* barriers in the overall KLIK implementation. These eight expert team members include all authors. When the majority (5 or more members) of the KLIK expert team identified a CFIR construct as barrier, this was entered in the matching tool. Specific agreement (both positive and negative, including 95% confidence intervals) was calculated according to De Vet et al. [33] using R.

Consequently, the tool provided output with percentages showing which ERIC implementation strategies can best be used to reduce these specific CFIR barriers. Percentages reflect the proportion of panelists endorsing a strategy appropriate for that barrier. Strategies are sorted by the cumulative percentage value. According to the tool, the strategies with the highest cumulative percentages are most effective in reducing the combined identified barriers [30]. In the results, the ten highest cumulative percentages, and, therefore, the overall advised strategies for the specific KLIK barriers will be shown. In addition, for every identified barrier using the CFIR-ERIC tool, the authors discussed what was already done in the past to reduce the impact of this barrier on the KLIK implementation process and the reasons why it still remains a barrier.

## Results

### A. Retrospectively describing the most prominent determinants of successful KLIK PROM implementation using CFIR

**Fig. 3:**
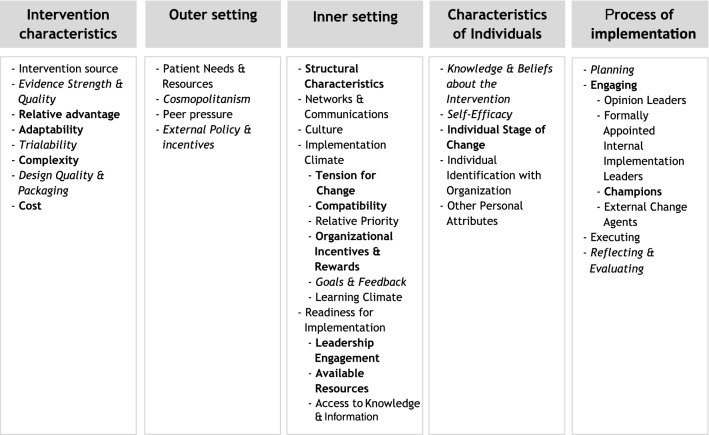
Overview of the five domains of CFIR, indicating determinants and barriers for the implementation of the KLIK PROM portal. Most prominent determinants are indicated in *italics*. The 13 identified current barriers using the CFIR-ERIC matching tool are indicated in **bold**

Based on previous research and on multiple years of experience implementing the KLIK PROM portal in clinical practice, the most prominent determinants were identified by the KLIK expert team (Fig. 3) and reasons for successful KLIK implementation are depicted in Table 1.

Several of the CFIR constructs were not applicable to the implementation of the KLIK PROM portal, unknown or differ too much between the different multidisciplinary teams and hospitals. These constructs include patient needs, networks & communications, culture, relative priority, learning climate, individual identification with organization, other personal attributes, and executing.

### B. CFIR-ERIC Implementation Strategy Matching Tool to identify *current* barriers of the KLIK PROM portal implementation

Of the 39 CFIR constructs, 13 were identified by the KLIK expert team as current barriers for implementing the PROM portal using the CFIR-ERIC matching tool. The total specific agreement was 68.1% (95% CI 59.6%–77.6%), positive agreement (CFIR barrier) was 75.9% (95% CI 68.1%–84.6%), and negative agreement (no CFIR barrier) was 53.1% (95% CI 44.0%–63.2%). In Table 2 and Fig. 3, the 13 barriers are shown. Per barrier is described what is already done as well as the challenges that remain.

**Table 2:** Identified *current* barriers for the KLIK PROM portal implementation using the CFIR-ERIC matching tool

CFIR domain	CFIR barrier	What is already done?	Why still a barrier?
Intervention characteristics	1. Relative advantage	Overall, clinicians working with the KLIK PROM portal think it is a valuable tool to monitor PROMs in their patients [12, 29, 35] The advantages about the KLIK PROM portal and discussing PROMs in the consultation room are spread during conferences and in scientific papers	Some stakeholders are reluctant to change and do not see the advantages of using PROMs, or suggest that alternative solutions (e.g., administering PROMs using EHRs or on paper) can be used
Intervention characteristics	2. Adaptability	KLIK is a very flexible system where many individual wishes of the multidisciplinary teams can be met (e.g., different PROMs for different patient groups at different timeslots, for different ages, various forms of feedback different for specific clinicians) To make KLIK as user friendly as possible. For example, KLIK is available in different languages and proxy reported PROMs are offered for patients with disabilities [13]	Clinicians prefer the intervention as tailored as possible. A standard set of PROMs is currently being offered to patients automatically based on age and patient group, not yet on an individual patient level (selecting specific PROMs per individual patient per visit)
Intervention characteristics	3. Complexity	The KLIK PROM portal is easy to use as a result of its origin in pediatrics. A recent evaluation study shows that the majority of clinicians (72%) think KLIK is easy to use [35] Together with the use of the KLIK PROM portal, hospitals receive support and advise from the KLIK expert team during all steps of the implementation (see Fig. 3)	For some clinicians KLIK remains complex to use, for example if they are not familiar with ICT. In addition, it requires additional actions, because clinicians need to actively motivate patients and sometimes send out the PROMs to their patients
Intervention characteristics	4. Cost	KLIK is being offered at low costs, as we are a non-profit organization, and alternative portals are often more expensive	Within healthcare there are often insufficient financial resources. Therefore, some teams still decide to refrain from using PROMs because of the additional costs
Inner setting	5. Structural characteristics	In general, KLIK is being implemented bottom-up, where small multidisciplinary teams show their interest in using KLIK	Hospitals are large organizations, and obtaining permission to change existing workflows can be a long process The board of the hospital might not be aware of bottom-up processes and can, therefore, be perceived as a barrier in larger scale implementation
Inner setting	6. Tension for change	Champions (clinicians who are enthusiastic about using KLIK) can explain the added value of using PROMs in clinical practice and persuade colleagues in trying out KLIK as well	Some clinicians do not see the current situation (not using PROMs in clinical practice) in a need of change
Inner setting	7. Compatibility	At the start of the KLIK implementation, the KLIK expert team advices on how to fit KLIK best into the existing workflow Recently, in four hospitals, a front-end integration with KLIK and the EHR (Epic© and HiX©) is realized	A study showed that a perceived barrier for stakeholders was compatibility (24% of clinicians indicated that the KLIK method did not fit well with current routines) [29]. To make it better fit with existing workflows, KLIK should be integrated into the EHR in all hospitals
Inner setting	8. Organizational incentives & rewards	Clearly communicate incentives (e.g., communication tool, improvement of quality of care, data can also be used for scientific purposes) of using PROMs in clinical practice for both patients and clinicians	Sometimes there are no incentives in the opinion of multidisciplinary teams and they, therefore, do not promote the use of PROMs in clinical practice
Inner setting	9. Leadership engagement	License agreements are signed by an authorized signatory and it therefore approves the implementation	Key organizational leaders or managers could show more commitment and involvement in KLIK by promoting it actively. In addition, in the current situation, they are not held accountable for implementation of the innovation
Inner setting	10. Available resources	KLIK has received several grants for the implementation and developed a business model to provide financial resources for the KLIK expert team in addition to the external resources	There is no structural funding yet for the KLIK expert team. To continue implementing KLIK, we are currently working on a new business model where we are not dependent on external funds, but can provide the use of KLIK at low costs For clinicians, money or time to discuss PROMs can be a barrier in implementing PROMs
Characteristics of individuals	11. Individual stage of change	When clinicians experience benefits from implementing PROMs, they become more enthusiastic By training clinicians, the skills necessary to implement and discuss PROMs are provided During the annual evaluation meetings we identify clinicians that do not perceive enough benefits or forget using KLIK. These meetings keep the clinicians focused on the goal of discussing PROMs From the perspective of patients, information letters, flyers, and educational videos are provided to give them the skills to complete and discuss PROMs. In addition, focus groups are held to explore their experiences regarding KLIK, in order to further optimize KLIK	Clinicians that do not feel skilled or enthusiastic about using the innovation in a sustained way are resistant to use the intervention. Feedback from patients includes that PROMs are not discussed by the clinician, they sometimes do not see the added value, and PROMs can be long and repetitive
Process of implementation	12. Champions	Most teams have a champion (an individual who support the KLIK implementation in a way that helps to overcome indifference of resistance by key stakeholders) who is motivated to start implementing KLIK for their patients	Some champions seem to have insufficient influence to convince their colleagues
Process of implementation	13. Engaging (Key stakeholders)	Clinicians are involved in the entire implementation process Also patients are more and more involved in the KLIK PROM portal, e.g., by asking their opinion in both qualitative and quantitative studies, developing educational videos to prepare them for the outpatient consultation, and by collaborating with patient associations	Patient engagement can be increased, for example, at the start of the implementation to explore the relevant PROs for patients

### Matching ERIC strategies to CFIR barriers

The identified barriers were matched to the 73 ERIC strategies using the CFIR-ERIC matching tool. Of these ERIC implementation strategies, the top 10 strategies matching the 13 identified CFIR barriers are shown in Table 3, sorted by the cumulative percentage value. Percentages reflect the proportion of panelists endorsing a strategy for that specific CFIR barrier. The tool shows that the strategy ‘identify and prepare champions’ is most effective in addressing the combination of identified barriers, followed by ‘promote adaptability’ and ‘assess for readiness and identify barriers and facilitators’.

**Table 3:** Output of the CFIR-ERIC matching tool: top 10 ERIC strategies matched to the 13 identified CFIR barriers for current KLIK implementation

CFIR barriers	Cumulative Percent	1. Relative advantage	2. Adaptability	3. Complexity	4. Cost	5. Structural Characteristics	6. Tension for Change	7. Compatibility	8. Organizational Incentives & Rewards	9. Leadership Engagement	10. Available Resources	11. Individual Stage of Change	12. Champions	13. Key Stakeholders
ERIC strategies														
Identify and prepare champions	449%	45%	23%	30%	12%	27%	48%	21%	25%	41%	4%	44%	67%	63%
Promote adaptability	312%	24%	73%	40%	16%	23%	17%	45%	4%	9%	4%	28%	11%	17%
Assess for readiness and identify barriers and facilitators	310%	24%	31%	30%	16%	36%	35%	34%	13%	14%	13%	12%	15%	38%
Alter incentive/ allowance structures	305%	28%	0%	7%	44%	18%	22%	10%	71%	32%	17%	32%	7%	17%
Conduct local consensus discussions	287%	24%	31%	7%	4%	14%	43%	41%	8%	27%	0%	20%	26%	42%
Inform local opinion leaders	261%	28%	15%	13%	12%	14%	39%	3%	17%	18%	0%	28%	44%	29%
Access new funding	226%	10%	0%	3%	72%	5%	0%	3%	38%	9%	78%	0%	4%	4%
Tailor strategies	218%	17%	35%	27%	12%	18%	13%	38%	17%	5%	9%	8%	4%	17%
Create a learning collaborative	218%	7%	23%	33%	8%	18%	9%	14%	13%	5%	9%	28%	19%	33%
Identify early adopters	217%	17%	27%	20%	8%	23%	13%	10%	13%	9%	0%	24%	41%	13%

Percentages shown reflect the proportion of panelists [30] endorsing a strategy appropriate for that barrier

## Discussion

This paper aimed to retrospectively describe the most prominent determinants of successful KLIK PROM portal implementation using the Consolidated Framework for Implementation Research (CFIR) and to identify current barriers and matching implementation strategies for the KLIK implementation using the CFIR-ERIC Implementation Strategy Matching Tool.

This retrospective evaluation shows that the strength of the KLIK PROM portal implementation lies particularly in the following CFIR domains: intervention characteristics (e.g., easy to use, direct feedback), characteristics of individuals (e.g., motivated clinicians), and process of implementation (e.g., support of the KLIK expert team). In addition, the climate of the outer setting is changing and patient-reported outcomes are more valued, which facilitates the implementation of the KLIK PROM portal. On the other hand, barriers in the implementation lie mainly in the domain of the inner setting and the intervention characteristics. Regarding the inner setting, involving and motivating all stakeholders at various levels of the multidisciplinary teams and hospitals is challenging. Regarding the intervention characteristics, mainly the tension field of providing optimal support of the KLIK expert team and the use of the KLIK PROM portal on the one hand and keeping low costs on the other hand is difficult. These findings are in line with another study discussing PROM implementation [38], where the authors describe the same relevant CFIR domains. This implies that the CFIR domain ‘outer setting’ might be less relevant than the other four domains when describing PROM implementation. However, a recent study on PREM implementation did find relevant outcomes regarding the outer setting, or macro level [39], and other literature on PROMs in palliative care also conclude that all CFIR domains need consideration for effective implementation [40].

Most CFIR domains were applicable to implementation of the KLIK PROM portal, showing that CFIR can be used in the context of implementing PROMs. However, the framework is not specifically developed for this context, resulting in insufficient attention for specific parts of the PROM implementation. For example, the content, length, and psychometric properties of PROMs are important factors for successful implementation of PROMs in clinical practice and are not addressed by the CFIR framework.

The CFIR is a comprehensive framework based on various published implementation theories [22], resulting in a very extensive framework consisting of many constructs, which can make it complicated to use. The five domains of the framework are intertwined and interacting, making it hard to determine where points of attention can be placed without iteration. In particular, the domain inner setting consists of many overlapping subdomains with intangible concepts. In addition, a recent systematic review on implementing e-health interventions shows blind spots in current literature about contextual factors (such as the organization), which makes it difficult for clinicians and researchers to understand these concepts and to translate it to clinical practice [41]. In previous literature, other weaknesses of CFIR are mentioned. In their systematic review on PROM implementation, Foster et al. identified the importance of different stages of the implementation process, which is not captured by CFIR [1].

The CFIR can be described as a determinant framework [24]. Determinant frameworks specify which factors (determinants) have a facilitating or inhibiting effect on the implementation. These frameworks thus describe the influence of processes on the implementation outcomes, but do not address these implementation outcomes, in contrary to evaluation frameworks. Therefore, it would be useful to use the CFIR in combination with another type of model. For instance, a widely used model on implementation outcomes is the “conceptual model of implementation research”, as described by Proctor and colleagues [18]. In order to improve outcomes for patients, it is important to be able to determine which determinants relate to which specific implementation outcomes. Only then can be reliably concluded which specific strategies work for which implementation outcomes.

The CFIR-ERIC Implementation Strategy Matching tool provided implementation strategies for the identified CFIR barriers [30]. Some of the suggested implementation strategies can be explored and used in the KLIK PROM portal implementation in the upcoming years. For example, assess key stakeholders for readiness is an ongoing process and still a challenge. By conducting individual interviews with the more reluctant clinicians underlying resistance can be better understand and addressed. In addition, identifying expected barriers and facilitators in the implementation process by actively discussing these topics in multidisciplinary team meetings in a more structured way is necessary. Also, incentives for patients in using the KLIK PROM portal could be explored further by increasing patient engagement.

However, not all suggested strategies by the matching tool provided new insights as they were directly linked to the perceived barrier (e.g., identify and prepare champions for the barrier ‘champions’ and access new funding for the barrier ‘cost’) and therefore were already known by the KLIK expert team. In addition, some strategies are currently being worked on (e.g., tailoring strategies, inform local opinion leaders, and identify barriers in the implementation process). Though, these strategies are difficult to implement and the tool underlines the need to pay more attention to these important strategies.

To further improve the KLIK implementation process in daily clinical practice, both the identified current barriers as well as the strategies extracted from the CFIR-ERIC tool can be used, to provide some examples:

- Recently, more and more evidence has become available for the *relative advantage* of implementing PROMs [42, 43]. We incorporate this information in the training to clinicians (step 4 in Fig. 2) and in the information we send to interested stakeholders to overcome this barrier. This might also affect the barrier *tension for change*.
- To overcome the barrier of *structural characteristics*, creating awareness within the board of hospitals to facilitate larger scale implementation can be an opportunity. This might also affect the barrier *leadership engagement*.
- Regarding *engaging key stakeholders*, patients and patient associations should be more involved in e.g., selecting PROs and PROMs and choices regarding frequency (step 1 in Fig. 2).

On the other hand, some current barriers will likely remain or even become more prominent in the future. For example *complexity*, due to increased privacy legislation, the KLIK PROM portal requires now the use of two-factor authentication, which does not benefit the usability of KLIK for some users.

At the time the implementation of the KLIK PROM portal in clinical practice started, a variety of implementation frameworks (including CFIR) and instruments to monitor and evaluate the implementation process from the start were not yet available. Just as we have evolved as a group, implementation science has evolved over the past decade as well. Implementation of the KLIK PROM portal was therefore essentially a process of “learning by doing”. Each time a specific multidisciplinary team showed interest in using KLIK, novel challenges appeared. As a result, a wide range of implementation strategies were used to tackle these particular issues. Notably, without realizing it at the time, many of the principles and strategies that are outlined in the CFIR tool were applied.

We recommend groups starting to implement PROMs in their setting to use an implementation science framework, like the CFIR, as knowing which factors need to be taken into account can lead to a more successful implementation in a specific context. The CFIR authors have developed an Interview Guide Tool (https://cfirguide.org/tools/) that can help researchers to question constructs of the CFIR that apply for the specific context. As every individual implementation process is different, also the constructs that are applicable differ.

Strengths of this study include the broad view of the retrospective description; multiple populations and multicenter experiences have been taken into account. In addition, the description is based on long-term experience and on published literature. However, this paper has several limitations. First, although a deliberate choice, no standardized qualitative research methods were used in this paper as the aim of this paper was to give a retrospective description of the KLIK PROM implementation process using the CFIR framework with the overarching purpose to create more awareness for the use of implementation science in PROM research. Second, the determinants and barriers for successful KLIK PROM implementation were described based on the experiences of the KLIK expert team (existing of members from two different centers) and this could have led to a selective view from the KLIK expert team. However, the KLIK expert team works closely with a variety of stakeholders on a day-to-day basis, including clinicians, patients, and parents. They furthermore provide opportunities for stakeholders to provide feedback during regular evaluation meetings. In addition, recently two evaluation studies were carried out to gain more insight into the perspectives of clinicians [35], and pediatric patients and parents [44]. Thus, even though other stakeholders were not literally represented as co-authors, it can be assumed that their opinions are represented throughout this study.

In conclusion, this retrospective approach showed that the CFIR provides clinicians and scientists guidance during a healthcare implementation process and can be used in all phases of implementation, although it is a quite extensive and complex framework with some overlapping constructs. For example, the CFIR can be used retrospectively, reflected in this article, to describe the implementation process and its determinants and to identify remaining barriers. An advantage of using this theoretical framework prior to start of implementation is that clinicians become aware of the possible facilitating determinants and barriers for implementation. Using an implementation science framework, like the CFIR, is recommended for groups starting to use PROMs in clinical care as knowing which factors need to be taken into account can lead to a more successful implementation in a specific context.

## Electronic supplementary material

Below is the link to the electronic supplementary material.

Supplementary file1 (DOCX 25 kb)
