# Supplementary material for: A retrospective assessment of the KLIK PROM portal implementation using the Consolidated Framework for Implementation Research (CFIR)
Source: Qual Life Res. 2020 Aug 15;30(11):3049–61. doi: 10.1007/s11136-020-02586-3 (PMC8528752; doi:10.1007/s11136-020-02586-3)
Supplement: Supplementary file 1 — Supplementary file1 (DOCX 25 kb) [file 11136_2020_2586_MOESM1_ESM.docx]

Supplemental table 1:

| **Authors** | **Year** | **Short title** | **Setting** | **Journal** | **Main outcomes** |
| --- | --- | --- | --- | --- | --- |
| Engelen et al [25] | 2010 | Development of QLIC-ON (predecessor of KLIK) | Pediatric oncology | Patient Educ Couns | A description of the development and implementation of the QLIC-ON Profile in pediatric oncology. |
| Haverman et al [28] | 2011 | Development of KLIK | Pediatric rheumatology | Pediatrics | The use of the KLIK ePROfile makes a positive contribution to systematic monitoring and discussing HRQOL issues in the consultation room. |
| Engelen et al [7] | 2012 | Effectiveness of KLIK | Pediatric oncology | Pediatr Blood Cancer | Using KLIK, emotional and psychosocial problems are more often discussed and identified during the consultation. |
| Engelen et al [45] | 2012 | Influences of PROs on discussion of psychosocial issues | Pediatric oncology | Pediatr Blood Cancer | The use of PROs increases the amount of psychosocial topics that are discussed during the consultation. |
| Haverman et al [12] | 2013 | Effectiveness of KLIK | Pediatric rheumatology | Pediatrics | The use of the KLIK ePROfile increased discussion about psychosocial topics and satisfaction of the clinician with the provided care. |
| Haverman et al [13] | 2014 | Implementation of KLIK | Pediatrics | Clin Pract Pediatr Psychol | The implementation of KLIK is feasible and workable. |
| Schepers et al [46] | 2014 | PROs in pediatric oncology: suggestions for future use | Pediatric oncology | Pediatr Blood Cancer | Parents and clinicians consider the use of PROs as an important part of standard care. |
| Santana et al [34] | 2015 | Training clinicians | Adult oncology, lung transplant and pediatrics | Qual Life Res | An important step in the implementation of PROs is training clinicians in how to use and act on PROMs in clinical practice. |
| Schepers et al [29] | 2017 | Real-world implementation of PROs | Pediatric oncology | Psycho-oncology | Implementing the KLIK PROM portal in pediatric oncology and a description of the barriers and enablers for this implementation process. |
| Schepers et al [47] | 2017 | Electronic feedback of the psychosocial assessment tool | Pediatric oncology | Support Care Cancer | Implementation of the electronic version of the psychosocial assessment tool seems feasible. |
| Schepers et al [48] | 2018 | The use of the electronic psychosocial assessment tool | Pediatric oncology | Cancer | Scores of the electronic psychosocial assessment tool at diagnosis are good predictors of parental stress in the future. |
| Haverman et al [36] | 2019 | Feedback options of PROMs in KLIK | Clinical practice | Med Care | Customization of the KLIK PROM portal is needed per patient group and per PROM. |
| Teela et al [35] |  | Experiences of clinicians with the use of KLIK | Clinical practice | Qual Life Res | Clinicians are generally satisfied with the usability of the KLIK PROM portal and the feedback of the KLIK ePROfile. |
| Van Muilekom et al [44] |  | Experiences of patients and parents with the use of KLIK |  | In progress | Patients and parents are satisfied with the use of the KLIK PROM portal. It helps them during the conversation with the clinician. |
